# Essential oil of *Cymbopogon martini*, source of geraniol, as a potential antibacterial agent against *Bacillus subtilis,* a pathogen of the bakery industry

**DOI:** 10.12688/f1000research.54196.1

**Published:** 2021-10-11

**Authors:** Sara Santamarta, A. Cristina Aldavero, M Angeles Rojo

**Affiliations:** 1 Area of Enginering and technology, Miguel de Cervantes European University, Valladolid, Castilla y Leon, 47012, Spain; 2 Area of Experimental Sciences, Miguel de Cervantes European University, Valladolid/Spain, Castilla y Leon, 47012, Spain

**Keywords:** Bacillus subtillis, Cymbopogon martinii, antibacterial activity, geraniol, rope disease, bakery industry

## Abstract

**Background:** Bacteria can adhere and grow on any surface due to their chemical and physical interaction, leading to the development of biofilms. Essential oils have a great potential for use in the food industry, as they can effectively prevent the presence of some pathogenic microorganisms.

Species such as those in the *Bacillus* genus have the ability to produce toxins. Some strains of *Bacillus subtilis* have been related to cases of food‐borne diseases. In the bakery industry, *B. subtilis* also has been related to “rope” disease, linked to bread preservation processes.

**Methods:** The aim of the study was to analyse the antibacterial properties of 24 chemotyped essential oils against the growth of *B. subtilis*. The biological activity study was carried out using disk diffusion in agar and broth dilution methods.

**Results:** The essential oil of *Cymbopogon martinii var. motia* had a high geraniol content (>80.53%) and showed a high antimicrobial effect against the Gram-positive bacterium *B. subtilis*. Binary combinations of *Cymbopogon martinii var. motia* oil with *Eugenia caryophyllus* showed antagonistic effects on *B. subtilis*.

**Conclusions: ** The essential oil of *Cymbopogon martinii var. motia* has an interesting potential use in the bakery industry as a preservative, in applications such as nano encapsulation for bakery doughs, active packaging of baked products, or surface disinfectants.

## Introduction

Essential oils (EOs) are aromatic and volatile natural compounds, synthesized and secreted by specialized histological structures. They are extracted from plant material, such as flowers, aerial parts, roots, bark, leaves and fruits. ^1^ EOs are secondary metabolites playing a role in the plant protection against biotic and abiotic stress. They constitute about 1% of plant secondary metabolites. ^2^ The composition of EOs from the same species of plant can vary with geographic location, the harvesting season, the part of the plant being distilled, or extraction method. ^3^^,^^4^

The chemical composition of EOs is complex; some of them have around 20 to 60 different bioactive components, only two or three of which are in concentrations within a range of 20% to 70%, with the rest found in trace amounts. Within this mix of bioactive components, two different compound groups can be identified: terpenes and terpenoids. Terpenes are the most abundant components in EOs, and are classified into monoterpenes and sesquiterpenes, according to their number of isoprene units. Within this group, α-pinene, β-caryophyllene, γ-terpinene, limonene, geraniol, and p-cymene are included. Monoterpenes can be linear (acyclic) or contain rings (monocyclic and bicyclic). Modified terpenes (containing oxygen molecules or lacking a methyl group) are called monoterpenoids, and include carvacrol, thymol, menthol, borneol, geranyl acetate, 1,8-cineole, and linalool. Terpenoids, present in the EOs (less predominant) are the aromatic compounds, derivatives of phenylpropane (mixtures of aldehydes, alcohols, phenols, methoxy derivatives and methylenedioxy compounds), for example, eugenol and cinnamaldehyde. ^5^^,^^6^

The mechanisms of EO antimicrobial action are mediated by a series of biochemical reactions and depends on the type on their chemical constituents. Gram-positive bacteria are not considered as resistant as Gram-negative bacteria. ^6^ This is attributed to differences in their cell wall structure, namely a thick peptidoglycan cell wall that allows penetration by phenolic compounds (for example, thymol, carvacrol, eugenol) present in EOs. ^7^ The hydrophobic characteristic of EOs grant them a greater accessibility to the cell wall of Gram-positive bacteria, which is rich in peptidoglycans and unable to resist the presence of small antimicrobial molecules, leading to variations in the structure of the cell membrane. Consequently, this leads to cell lysis and leakage of intracellular compounds. ^8^ These changes in the permeability of the cell wall and cytoplasmic membrane affect bacterial spread. Bacteria, in the presence of antimicrobial agents, alter the lipid profile of their membrane to incorporate exogenous fatty acids (from EOs), modifying the ratio of fatty acids, or the length of their carbon chains, and can increase the amount of saturated, or decrease the amount of unsaturated fatty acids. This reorganisation of fatty acids and membrane proteins allows for the survival of bacterial cells. ^6^ However, the synergistic action of different EO constituents favours the death of the bacterial cell. ^9^^,^^10^

The use of some EOs has been considered as an antimicrobial alternative, and has attracted considerable interest from the pharmaceutical industry, especially for its antifungal and antiviral activity. ^11^^–^^14^ Phytochemicals present in EOs are also being evaluated as inhibitors of COVID-19. ^15^ In the food industry, EOs are used for food preservation, due to their natural antimicrobial compounds against pathogenic bacteria, as well as aroma and flavour. ^16^^,^^17^

The *Bacillus* specie *s* are included among Gram-positive bacteria. They are aerobic or facultative anaerobic bacteria that are widely distributed in nature. ^18^ *B. subtilis* is considered as an aerobe bacterium, although it is able to sporulate under anaerobic conditions and starts to proliferate and grow in different environments including water, processed and untreated foods. ^19^ Nowadays, the bakery industry is growing, giving rise to an increased demand for quality, new products developed, and for the extension of products’ shelf life and safety. Previous studies have considered the use of EOs as antifungals to prolong bread shelf life. ^20^ Research results from Pepe *et al.* (2003) ^21^ have determined that after a 10-minute treatment at 96°, *Bacillus* spp. were able to survive the thermal processing of bakery products. Contamination of the dough by spores can occur via flour or the reuse of dried and ground bakery. ^22^ The prevalence of spore-forming string-producing microorganisms in different types of flour and their potential for bread spoilage has been investigated; *Bacillus* spp. spores exhibit a high heat resistance and regularly survive the baking process inside the bread. Due to the action of proteolytic and amylolytic enzymes released by *Bacillus*, the texture of the bread is modified and becomes viscous. ^22^ One of the species considered to be the most common causative agent of rope in bakery products is *B. subtilis*, ^23^ however, as it takes a relatively long time for the stiffness to develop, the deterioration is often only detected once the consumer has purchased the product. There is increasing interest in developing new strategies to inactivate spores. EOs contained into microcapsules that gradually release volatile compounds into the packaging environment have been used in food preservation. ^24^^–^^26^ Still, there is little information on the application of EO-containing microcapsules in the preservation of baked products.

The aim of this study was to evaluate the effect of 24 chemotyped essential oils on *Bacillus subtilis* growth and spore production, for potential use in the bakery industry, to answer a growing demand for high quality and extended shelf-life products, and the increasing trend of consumer demand for clean labels and minimum processing or synthetic preservatives. EOs were chosen based on their commercial availability, scent, and absence of toxicity in view of a possible bakery industry use. All of them are used traditionally as food additives. Of all EOs, *Cymbopogon martinii var. motia* was found to be an important source of natural geraniol. According to the literature review carried out by Lira *et al.* (2020), ^27^ geraniol presents many pharmacological properties including antifungal and antibacterial actions. A secondary objective was to investigate the combined antibacterial activities of *Cymbopogon martinii var. motia* (palmarosa oil), *Mentha x piperita* (peppermint) and *Eugenia caryophyllus* (clove) essential oils against *B. subtilis.*

## Methods

### Bacterial strains and media

*Bacillus subtilis* subsp. *subtilis* (Ehrenberg 1835) Cohn 1872, from the SpanishType Culture Collection (CECT), listed as CECT 4522, was used in this study. *B. subtilis* was maintained on a nutrient broth medium and solidified. The growth temperature was 30°C and the incubation time was 24 h. The nutrient broth medium contained 0.5% of beef extract (Laboratorios Condalab S.L., Madrid, Spain), 1% of peptone (Laboratorios Condalab S.L., Madrid, Spain) and 0.5% of NaCl (Merck Life Science S. L, Madrid, Spain); after mixing and dissolving them completely, the medium was adjusted to pH 7.2.

### Essential oils

Chemotyped EOs were extracted from different plants by steam distillation by the supplier before purchase (Pranarôm, S.A., 7822, Ath, HAINAUT Belgium). The 24 EOs used for the study were: *Citrus sinensis* (sweet orange) *, Citrus reticulata* (Mandarin orange) *, Elettaria cardamomum* (cardamom) *, Laurus nobilis* (laurel) *, Cymbopogon martinii var. motia* (palmarosa) *, Zingiber officinale* (ginger) *, Eugenia caryophyllus* (clove) *, Cinnamomum camphora* (camphor tree) *, Rosmarinus officinalis* (rosemary) *, Melaleuca quinquenervia* (niaouli) *, Chamaemelum nobile* (Roman chamomile) *, Melaleuca alternifolia* (tea tree) *, Thymus vulgaris CT LINALOL* (thyme) *, Citrus paradisi* (grapefruit) *, Citrus junos* (yuzu) *, Origanum compactum* (oregano) *, Mentha x piperita* (peppermint) *, Myrtus communis* (myrtle) *, Curcuma longa* (curcuma) *, Cinnamomum cassia* (Chinese cinnamon) *, Thymus satureioides* (savoury thyme) *, Eucalyptus radiata* (eucalyptus) *, Cinnamosma fragrans (saro)* and *Mentha arvensis* (wild mint) *.* Their chemical components are shown in Table 1.

**Table 1.** Chemical components from the essential oils. The table shows the essential oils analyzed: common name, part of the plant and a percentage of some chemical components of selected essential oil after being subjected to steam distillation (data obtained from analysis sheet of Pranarôm; https://pranarom.us/products/essential-oils/).

Essential oil	Common name	Part subjected to steam distillation	Chemical components of selected essential oil constituents (%)
*Chamaemelum nobile*	Roman Chamomile	Flower.	Methylamine angelate (20.2%); Metalyl angelate (15.4%); Hexyl isobutyrate (8.31%)
*Cinnamomum camphora*	Ravintsara	Leaves.	1,8-Cineole (56.8%); Sabinene (13.4%); α-Terpineol (7.33%)
*Cinnamomum cassia*	Chinese canceller	Bark and leaves.	E-Cinnamaldehyde (81%), Cinnamyl acetate (3.25%)
*Cinnamosma fragrans*	Saro or Mandravasarota	Leaves.	1,8-Cineole (37.7%), Linalool (8.03%), Limonene (7.83%)
*Citrus junos*	Yuzu junos	Shell.	Limonene (75.6%); γ-Terpinene (8.49%); β-Phellandrene (3.29%)
*Citrus paradisi*	Grapefruit	Shell.	Limonene (94.5%)
*Citrus reticulata*	Mandarin	Shell.	Limonene (71.1%); γ-Terpinene (18.3%)
*Citrus sinensis*	Sweet Orange	Shell.	Limonene (95.3%)
*Curcuma longa*	Safran from india	Rhyzome.	α-Turmerone (34.8%), β-Turmerone (16.16%), Curlone (16.2%), AR-turmerone (13.4%)
*Cymbopogon matinii* var.motia	Palmarosa	Aerial part.	Geraniol (80.5%); Geranyl acetate (8.95%); Linalool (2.45%); β-Caryophyllene (1.87%)
*Elettaria cardamomum*	Cardamon	Fruit.	α-Terphenyl acetate (35.3%); 1,8 Cineole (32.3%); Linalyl acetate (5.35%); Linalool (3.35%)
*Eucalyptus radiata* ssp *radiata*	*Eucalyptus officinally*	Leaves.	1,8-Cineole (66.6%), α-Terpineol (11.2%), Limonene (6.5%)
*Eugenia caryophyllus*	Clove Bud	Flower bud.	Eugenol (79.9%); Eugenyl acetate (12.3%); β-Caryophyllene (5.39%)
*Laurus nobilis*	Laurel Noble	Leaves.	1,8 Cineole (44.9%); Terphenyl acetate (10.5%); Sabinene (8.86%); Linanool (4.43%)
*Melaleuca alternofolia*	Tea Tree	Leave.	Terpinene 4-ol (40.6%); γ-Terpinene (21%)
*Melaleuca quinquenervia*	Niaouli	Flowery peak.	1,8-cineole (50.6%); α-Terpineole (8.91%); Limonene (7.48%)
*Mentha arvensis*	Field mint	Aerial part.	Menthol (71.1%); Menthone (5.88%); Isomenthone (3.85%); Limonene (2.52)
*Mentha x piperita*	Pepper mint	Aerial part.	Menthol (44.5%); Menthone (18.2%); 1,8 cineole (4.64%)
*Myrtus communis*	Cineole blueberry	Leafy Branch.	α-Pinene (51%), 1,8 Cineole (22.8%), Limonene (8.34%)
*Origanum compactum*	Origan compact	Flowery peak.	Carvacrol (57.6%); Thymol (8.21%); γ-Terpinene (14.1%)
*Rosmarinus officinalis*	Verbenone Rosemary	Flowery peak.	α-Pinene (38.8%); Camphene (8.88%); Camphor (6.96%); Bornyle acetate (6.94%)
*Thymus satureioides*	Thyme with Savory Leaves	Flowery peak.	Borneol (33.4%); Thymol (10.6%); Carvacrol (7.85%); β-Caryophyllene (5.82%)
*Thymus vulgaris CT Linalol*	Cimbru	Flowery peak.	Linalool (68.4%); Linalyl acetate (6.19%); β-myrcene (3.32%)
*Zingiber officinale*	Ginger	Rhyzomes.	α-Zingiberene (28.2%); α-Curcumin (7.93%); Camphene (7.9%); β-Sesquiphelandrene (7.56%)

### Antibacterial activities of essential oils

The assessment of the antibacterial activities of EOs was performed using the disk diffusion method. ^28^ Bacterial inoculum was measured using a spectrophotometer (Libra S12, Biochrom Ltd, Cambridge CB4 0FJ, England) set at 600 nm, and 0.20 mL at DO 0.45 were transferred to the plates *.*

The disk absorption capacity was 5 μL/disk. Three different concentrations of EO extracts were aseptically transferred to these disks to establish their antimicrobial activity. Sterile disks were impregnated with 5 μl of EO at different concentrations by serial dilution in vegetal oil (100%, 10%, 1%) (v/v) and each disk was placed on a nutrient broth agar plate smeared with *B. subtilis.* Every dish was sealed with laboratory film to avoid evaporation, then incubated aerobically in an upright position at 30 °C for 24 h to determine the antimicrobial effect. Antibacterial activity was determined by measuring the inhibition zone diameter (mm) against each EO. A sterile vegetal oil without EO was used as a negative control, and 5 μg of Ciprofloxacin (Thermo Fisher Scientific, Waltham, MA, USA) as positive control (dc). ^29^ All experiments were done in triplicates *.*

The percentage of bacterial growth inhibition of *B. subtilis* was determined by considering the diameter of inhibition bacterial growth with EOs (dEO) referenced to the diameter inhibition bacterial growth with Ciprofloxacin antibiotic (dCIP), according to the following equation ( Equation 1)

$$\%\;\text{of}\;B.\text{subtillis}\;\text{growth}=\frac{\left(\text{dCIP}-\text{dEO}\right)}{\text{dCIP}}\;100$$ (1)

### Determination of minimum inhibitory concentration of essential oils against *B. subtilis*

Once the results of the antimicrobial activity against *B. subtilis*, obtained by disk diffusion, were analysed for the 24 EOs tested, ten EOs were selected ( *C. cassia, C. sinensis, C. martinii var. motia, E. cardamomum, E. caryophyllus, M. x piperita, T. vulgaris CT LINALOL and T. satureioides).* This selection also considered the principal chemical component in their composition. A *B. subtilis* inoculum (DO _600_ = 0.350) was incubated into the tubes containing different concentrations of these selected EOs (10 μL/mL, 25 μL/mL, 50 μL/mL, and 75 μL/mL). The tubes were incubated at 30 °C for 24 h and bacterial growth was measured using a spectrophotometer (Libra S12, Biochrom Ltd, Cambridge CB4 0FJ, England) at 600 nm. ^30^ Vegetal oil was tested as negative control, at the same volume as the EOs.

The percentage of *B. subtilis* bacterial growth inhibition was determined by considering the optical density of bacterial growth without EOs (ODc) minus the optical density of bacterial growth with the presence of EOs (ODp), divided by the optical density of bacterial growth without EOs (ODc) ( Equation 2).

$$\%\;\text{of}\;B.\;\text{subtillis}\;\text{growth inhibition}=\frac{\left(\text{ODc}-\text{ODp}\right)}{\text{ODc}}\;100$$ (2)

### Combined antibacterial effect of EOs using disk diffusion tests

The interaction studies (I) of the major components of EOs were performed using the agar disk diffusion method. The binary mix 1:1 (v/v) of EOs or their components, absorbed on sterile paper disks (5 μL per Whatman disk of 5 mm diameter), were placed on the surface of media that had previously been inoculated with 200 μL of *B. subtilis* (DO _600_ = 0.45). One filter paper disk was placed in each Petri dish, which was sealed with laboratory film to avoid evaporation, then incubated aerobically at 30°C for 24 h, followed by measurements of the diameter of the inhibition zone in cm.

The interaction was calculated as IA + IB, where IA and IB are the diameter of the inhibition zone (D) for EOs A and B, respectively. Thus, it was calculated as follows: IA = (DA combination / DA alone) and IB = (DB combination/DB alone). The results were interpreted as antagonism (I < 0.5) or indifference (0.5 ≤ I ≤ 1). All experiments were done in triplicates.

### Statistical analyses

Data were recorded using Microsoft Excel, which was also used for graphic data representation. Statistical analysis was performed using SPSS v27.0 software (SPSS Inc., Chicago, IL, USA). Normality of the dependent variables was checked using the Shapiro-Wilk test. If normality was observed, a one-way analysis of variance (ANOVA) was carried out. If normality was not observed, the Kruskal-Wallis test was used. The variability among the three measures was reported with the coefficient of variation. The reliability of the three measures ( *Underlying data*) was reported with the intraclass correlation coefficient (2.1) looking for absolute agreement. A significance level of 0.05 was used. Values are reported as the mean ± standard deviation (SD).

## Results

### Chemical composition of EOs (Pranarôm [S.A])

EOs are complex mixtures of volatile compounds; their main components are described in Table 1. The information was obtained from a data sheet provided by delegates from Pranarôm, S. A (Spain) and available on the Pranarôm website. As shown in Table 2, these volatile molecules include terpenes (hydrocarbon and oxygenated monoterpenes), terpenoid (oxygen atoms added to the hydrocarbon molecules), phenylpropene (phenyl group attached to an unsaturated aldehyde or ether), alcohol terpene (saturated secondary alcohol) and sesquiterpenes (hydrocarbon and oxygenated sesquiterpenes).

**Table 2.** Antibacterial activity of essential oils by the agar diffusion disk method. The essential oils have been grouped into five classes of considering the chemical structure of their major component.

Class	Essential oil	Diameter of inhibition zone *B. subtilis* growth (cm) ^1^			% *B. subtilis* growth inhibition against Ciprofloxacin		
		100%	10 %	1%	100%	10%	1%
Terpene	*Cinnamomum camphora*	1.7 ± 0.3	1.0 ± 0.1	0.2 ± 0.3	58.95	75.23	95.74
	*Cinnamosma fragrans*	1.2 ± 0.5	0.2 ± 0.3	0.2 ± 0.3	70.04	95.23	96.12
	*Citrus junos*	1.6 ± 0.4	0.5 ± 0.4	0.2 ± 0.3	61.71	88.75	95.74
	*Citrus paradisi*	1 ± 0.2	0 ± 0	0.2 ± 0.3	76.80	100	95,35
	*Citrus reticula*	1 ± 0.3	0.2 ± 0.3	0 ± 0	74.77	95.24	100
	*Citrus sinensis*	1.6 ± 0.3	0.5 ± 0.4	0.4 ± 0.4	61.63	87.96	90.70
	*Cymbopogon matinii var. motia*	2.4 ± 0.9	0.9 ± 0.3	0.2 ± 0.4	40.25	77.83	94.96
	*Elettaria cardamomum*	2.1 ± 0.3	0.7 ± 0.3	0.4 ± 0.3	48.01	83.98	91.47
	*Eucalyptus radiata ssp radiata*	1.2 ± 0.3	0,3 ± 0.3	0 ± 0	70.72	93.65	100
	*Laurus nobilis*	1.5 ± 0.2	1.0 ± 0.3	0 ± 0	63.22	74.58	100
	*Melaleuca alternofolia*	1.2 ± 0.1	0.2 ± 0.4	0 ± 0	69.70	94.44	100
	*Melaleuca quinquenervia*	1.5 ± 0.1	0.7 ± 0.2	0.4 ± 0.4	64.39	81.90	89.92
	*Myrtus communis*	1.1 ± 0.2	0 ± 0	0 ± 0	72.21	100	100
	*Rosmarinus officinalis*	1.3 ± 0.2	0.9 ± 0.3	0.2 ± 0.4	68.53	78.30	94.57
Terpenoid	*Chamaemelum nobile*	1.3 ± 0.2	0.4 ± 0.4	0 ± 0	68.55	90.35	100
	*Origanum compactum*	4.1 ± 0.1	1.1 ± 0.2	0.7 ± 0.1	0	72.83	84.11
	*Thymus vulgaris CT Linanol*	1.5 ± 0.3	0.9 ± 0.3	0.5 ± 0.5	62.95	77.94	87.60
	*Thymus satureioides*	2.4 ± 0.5	0.7 ± 0.2	0.3 ± 0.3	40.50	83.14	92.25
Sesquiterpene	*Curcuma longa*	1.2 ± 0.3	0.6 ± 0.1	0.2 ± 0.3	71.53	84.30	95.74
	*Zingiber officinale*	1.1 ± 0.4	0.5 ± 0.4	0 ± 0	74.04	88.75	100
Phenylpropene	*Eugenia caryophyllus*	1.7 ± 0.3	0.7 ± 0.2	0 ± 0	57.61	83.61	100
	*Cinnamomum cassia*	4.1 ± 0.1	1.6 ± 0.8	0.9 ± 0.2	0.77	59.89	79.07
Terpene alcohol	*Mentha arvensis*	1.7 ± 0.1	0.4 ± 0.3	0.2 ± 0.3	57.38	91.17	96.12
	*Mentha x piperita*	1.6 ± 0.4	0.7 ± 0.3	0.3 ± 0.5	60.08	82.03	93.02

^1^ Values are mean diameter of inhibitory zone (cm) ±SD of three replicates. The diameter of paper disk (0.5 cm) is included.

In the terpenes analysed, limonene, a monocyclic monoterpene, was present in *Citrus junos, C. paradisi, C. reticula* and *C. sinensis;* γ-terpinene was present in *C. junos* and *C. reticula*, highlighting the presence of β-phellandrene in *C. junos.* Geraniol was the most abundant compound in the EO of *C. matinii var. motia,* along with a significant percentage of geranyl acetate, and the presence of a bicyclic sesquiterpene, β-caryophyllene, as well as linalool (terpene with an alcohol group). The ether monoterpene 1,8-cineole, was present at a high percentage in *C. camphora, C. fragrans, E. radiata, L. nobilis, E. cardamomum*, *M. quinquenervia* and *M. communis.* Terpinene-4-ol and ʏ-terpinene, were identified in *M. alternofolia*, while *Rosmarinus officinalis* was characterized by α-pinene, followed by camphene, camphor and bornyl acetate. *M. communis* contained monoterpenes such as α-pinene, 1,8-cineole and limonene.

Among the main terpenoids identified in the EOs of *O. compactum* and *T. satureioides* were carvacrol and thymol; in contrast, borneol and β-caryophyllene were only present in *T. satureioides.* *Thymus vulgaris* contained linalool while and linalyl acetate. The main constituents of *C. nobile* were methylamine angelate, metalyl angelate and hexyl-isobutyrate.

Major components of *C. longa* belong to the chemical class sesquiterpenes which includes α-turmerone, β-turmerone, curlone and AR-turmerone. In addition, within this group, zingiberene is a monocyclic sesquiterpene and the predominant constituent of the oil of *Z. officinale.* Eugenol, hydroxyphenyl propene, was present in the essential oils of *E. caryophyllus.* Cinnamaldehyde is an aromatic aldehyde and main component of bark and leaf extract of *C. fragrans.* An additional terpene alcohol analysed belonged to *M. x piperita* and *M. arvensis.* The major chemical constituent of *M. x piperita* were menthol, menthone and 1,8 cineole, which are all cyclic monoterpenes. The main difference between these molecules is the presence of different functional groups: alcohol, ketone, and ether respectively. In addition, the difference with the EO of *M. arvensis* was the presence of isomenthone and limonene instead of 1,8-cineole.

### Antimicrobial activity

The results of this study show that not all tested EOs had the same activity against the growth of *B. subtilis.* Using the disk diffusion method, the Gram-positive bacteria under study was inhibited by all the citrus oils tested ( Table 2), which confirms previous works. ^31^^,^^32^ When comparing these terpene EOs’ antimicrobial activity using the agar-disk diffusion assay, we observed that the effectiveness of limonene action increased when other terpenes were mixed, which is evidenced by contrasting the inhibitory results against the bacteria from the action of *Citrus sinensis* and *C. junos.* Although there is different concentration of limonene in both EOs ( Table 1). These synergistic antimicrobial effects of the isolated compounds from *C. limon* against Gram-positive bacteria and *B. subtilis also* was observed by Nsangou *et al.* (2021). ^33^

*C. martinii var. motia* exhibited the second most potent antibacterial activity after *O. compactum,* among all tested EOs ( Table 2). The antibacterial activity of *O. compactum* has been related to the presence of two aromatic terpenoids: carvacrol and thymol ^34^ as opposed to geraniol, an acyclic monoterpene alcohol, ^27^ the main ingredient of *C. matinii var motia* ( Table 1).

We observed the antimicrobial activity of seven EOs ( *C. camphora, C. fragrans, E. radiata, L. nobilis, E. cardamomum, M. quinquenervia* and *M. communis*) in which the terpene 1,8-cineole ( Table 2) was present in relatively high concentrations. *E. cardamomum* showed the greatest inhibitory effect. A similar antimicrobial activity on the agar disks was observed for *C. camphora, L. nobilis and M. quinquenervia.* The difference in the antimicrobial activity between them may be due to the presence of some terpenes in their composition and their synergistic effects. ^36^ For example, *E. cardamomum, L. nobilis* and *C. camphora* showed a greater antimicrobial activity, with the former containing α-Terpenyl acetate in greater proportions than *L. nobilis*, and *C. camphora* containing sabine ( Table 1) *.*

The remarkable difference between the chemical composition of *M. alternofolia* and *M. quinquenervia,* and their similar effect against the growth of *B.subtilis,* may be due to the presence of terpinene 4-ol ^37^ in the former, and the presence of 1,8-cineole in the latter. The chemical components α-pinene and bicyclic terpenes could be found in the essential oil of *Rosmarinus officinalis* ( Table 1) *,* whose antimicrobial activity was greater than that of *Myrtus communis.* Rosemary oil also contained other major components, such as camphene, camphor and bornyle acetate ^37^; their synergistic effect gives Rosemary oil a greater antimicrobial activity. ^38^

In an agar-disk diffusion assay ( Table 2), cinnamaldehyde from *Cinnamomum cassia* (Chinese cinnamon oil) showed a higher antimicrobial activity against *B. subtilis* than eugenol from *Eugenia caryophyllus* (clove oil) *.* As presented in Table 2, the EO of *Cinnamomum cassia* showed an antibacterial activity comparable to that of *Origanum compactum* oil, preventing the growth of bacteria *.* According to the results reported in Table 2, it seems that the synergistic action of carvacrol and thymol from *O. compactum* is comparable to the activity of cinnamaldehyde.

The terpenoid group included *T. vulgaris* and *T. satureioides* oils. The former contained linalool ( Table 1), which from the inhibition zone showed less inhibitory activity than *T. satureioides* ( Table 2) *.* The action of borneol present in *T. satureioides* led to a greater growth inhibition of the bacteria through the synergistic action of carvacrol and thymol ( Table 2) present in *O. compactum.* These bioassays by disk diffusion showed a similar inhibition growth activity in *O. compactum* and *Cinnamomum cassia* EOs *.*

Menthol, the dominant compound present in *M. arvensis* and *M. x piperita oil* ( Table 1), showed a moderate antibacterial activity against *B. subtilis* growth ( Table 2) using the disk diffusion method *.*

Most of the undiluted, commercially available EOs used, gave rise to an inhibition zone against *B. subtilis.* Among the 24 oils tested, it was observed that their antibacterial effects were reduced at lower oil concentrations, suggesting that the inhibition halo was dependent on each essential oil.

**Figure 1.**
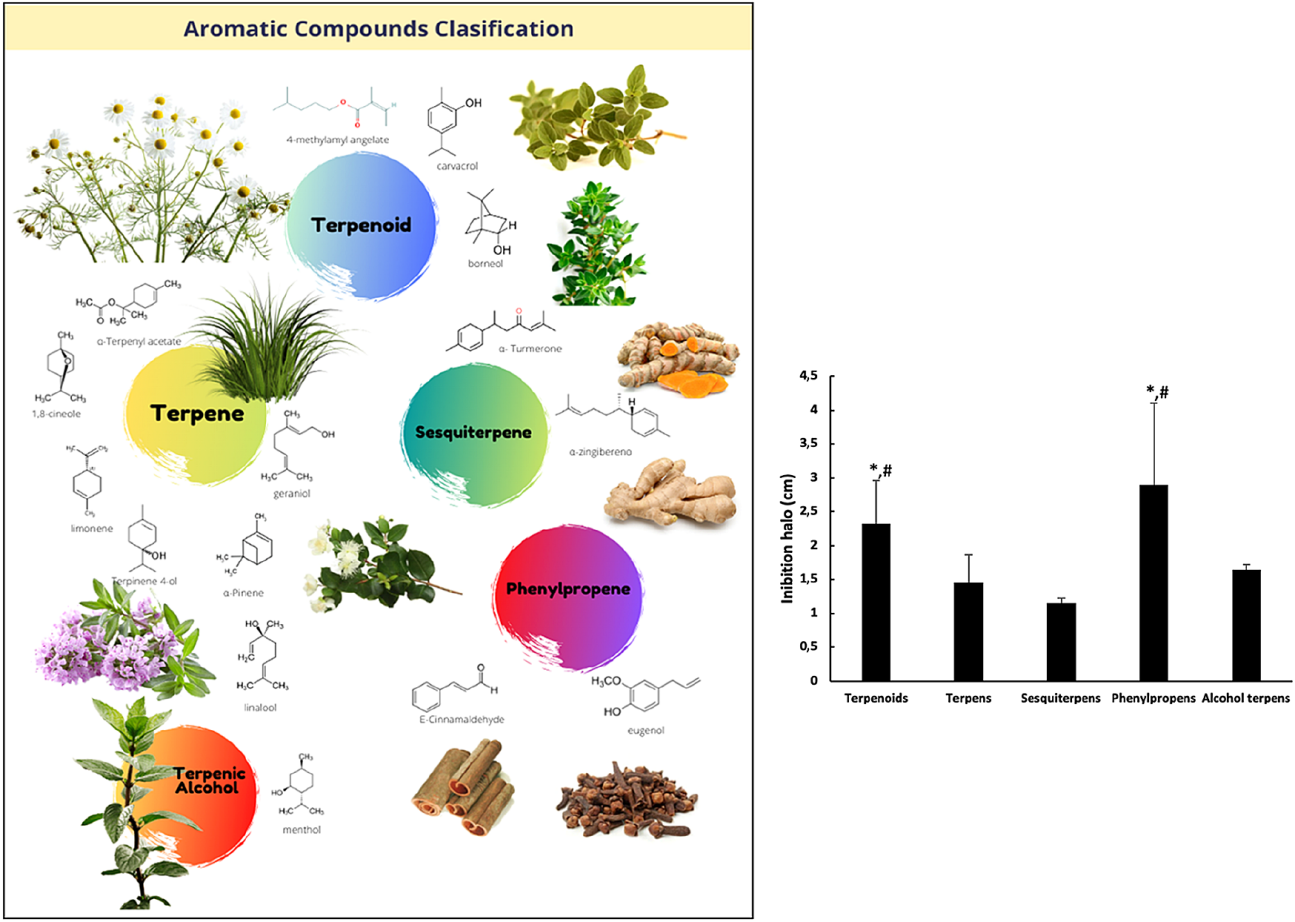
Effect of chemical components of essential oil constituents on the growth of *B. subtilis.* In the left side, the five chemical components groups of the EOs under study. In the right side, the graphical corresponding to the medium values of agar diffusion disk activity against *B. subtilis* growth for each EOs group. (*) differences with respect to terpenes with p < 0.05; (#) differences with respect to sesquiterpenes and alcohol-terpenes with p < 0.05.

The antimicrobial action of these EOs, as shown by agar-disk diffusion tests, was comparable to the activity of the antibacterial ciprofloxacin ( Table 2), while the vegetal oil control did not affect the growth of bacteria. The assessed antibiotic ciprofloxacin was selected for its antibacterial effect, previously described by Citron and Appleman (2006) ^29^ showing antimicrobial action against *B. subtilis* spores. To this end, EOs were arranged into five groups according to their main chemical component responsible for their inhibitory activity: terpen, terpenoid, alcohol terpenoids, sesquiterpenes and phenylpropanoids ( Figure 1A). Figure 1B shows the activity of chemical phenolic groups with respect to the other chemical groups. These results showed the following order in antimicrobial efficiency in agar diffusion disk assays: phenylpropene > terpenoid > alcohol terpene > terpene > sesquiterpene, without reaching statistical significance. It was found that the oils containing alcohol, ketone, ester, oxide, and hydrocarbon as major constituents showed high antimicrobial activity, but even higher antimicrobial activity was found in the oils containing phenol or phenyl derivatives that contain aldehyde and methoxy groups exhibited the highest antibacterial activity. This activity was previously observed by other authors. ^6^^,^^39^ Table 3 shows the reliability, referring to the consistency of the three diameter measures, relating to the zone of growth inhibition of *B. subtilis* (H), and the data when comparing the activity of the 24 EOs at three different concentrations, with the inhibitory action of Ciprofloxacin (HA) against the same bacterium. Reliability values closer to 1 represent a stronger reliability, however, it decreases as EOs are diluted.

**Table 3.** Values of reliability. Values of reliability from three replicates of measures of the diameter of inhibitory zone *B. subtilis* growth (H) and the activity respect to the action of antibiotic Ciprofloxacin (HA) of the 24 EOS at three different concentration.

	EOs concentration		
	100%	10%	1%
H	0.963	0.773	0.616
HA	0.954	0.802	0.620

**Figure 2.**
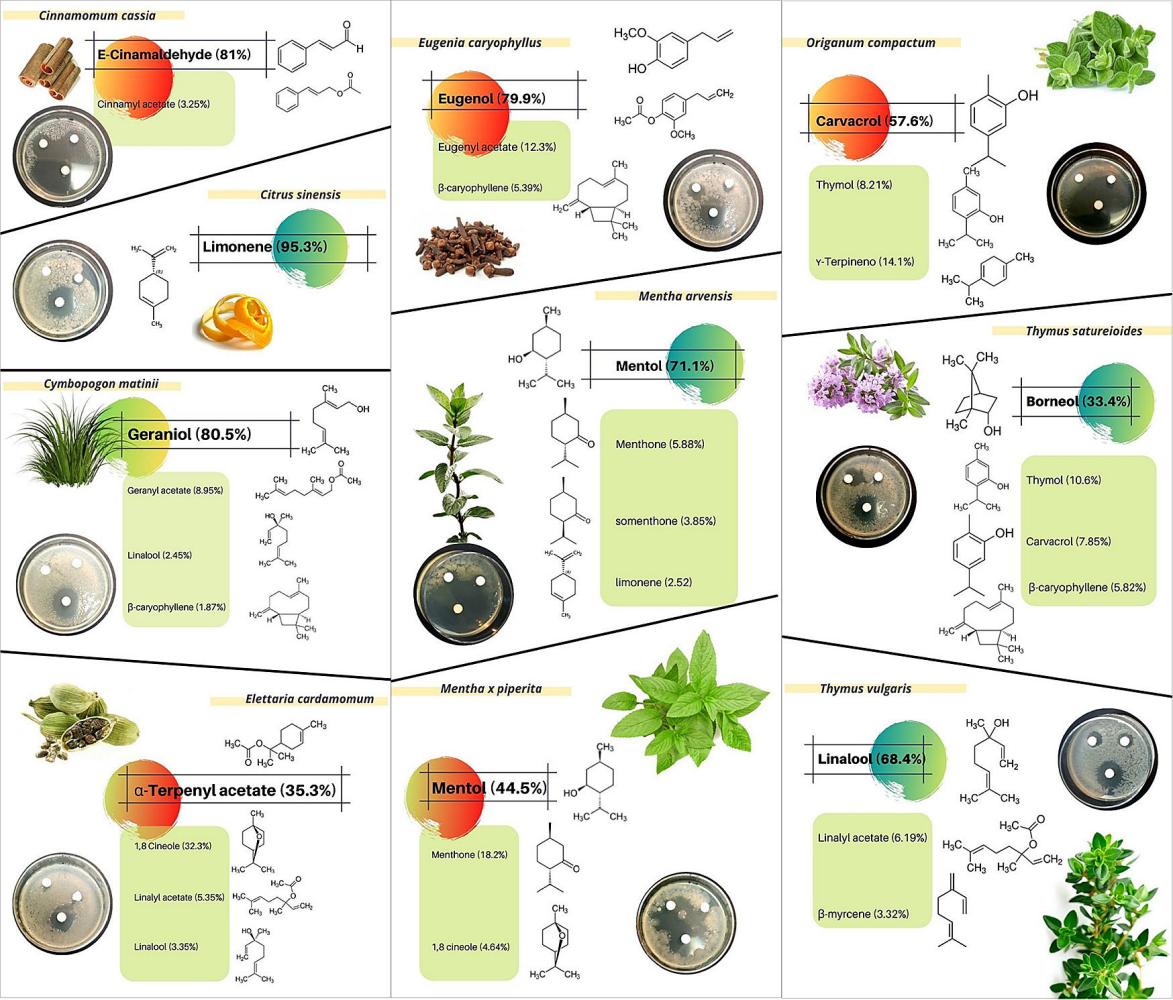
Antimicrobial activity of *Cymbopogon matinii var. motia*, *Elettaria cardamomum, Eugenia caryophyllus, Mentha x piperita, Thymus vulgaris CT Linanol* and *Thymus satureioides* by the diffusion method against *Bacillus subtilis.* Likewise, the major and biologically bioactive constituents present in the six essential oils are shown.

The results obtained in this study from inhibitory diameters and concentrations of 24 EOs, revealed that ten of them showed higher inhibition against *B. subtilis* growth ( Figure 2): *Cinnamomum cassia* (Chinese cinnamon) *, Citrus sinensis* (sweet orange) *, Cymbopogon matinii var. motia* (palmarosa), *Elettaria cardamomum* (cardamon) *, Eugenia caryophyllus* (clove bud) *, Mentha arvensis* (field mint) *, Mentha x piperita* (pepper mint) *, Origanum compactum* (oregano) *, Thymus vulgaris CT Linanol* (thyme) and *Thymus satureioides* (thyme with savoury leaves). These EOs represent four of the five earlier described groups ( Table 2); each of them containing different chemical components found to have high antimicrobiological action by Lira *et al*. (2020), ^27^ Fisher and Phillips (2006), ^32^ Laghmouchi *et al.* (2018), ^34^ Mulyaningsih *et al.* (2010) ^35^ and Bassolé and Juliani (2012). ^40^ To analyse their antimicrobial activity in aqueous solutions, the Minimum Inhibitory Concentration (MIC) of EOs was determined as described in our Methods. Table 4 shows the inhibitory activity of ten EOs against the growth *B. subtilis,* at four different concentrations (v/v) in nutrient broth culture. The lowest MIC values were found for cardamon, sweet orange, field mint and palmarosa oils. The lowest antimicrobial activities were observed for thymus, Moroccan thyme, peppermint and clove bud oil. The activity of Chinese cinnamon and compact oregano oil in aqueous solutions were lower than that observed using the diffusion method, where the EO is laid on a paper disk. After a 24 h-incubation period of *B. subtilis* in the presence of Origanum compactum oil, a colour change was observed in the culture broth medium; this may be due to the presence of carvacrol, which increases permeability of the bacterial membranes and releases H ^+^ into the culture medium. ^6^^,^^41^

**Table 4.** Minimum Inhibitory Concentration (MIC) values of some essential oils against *B. subtilis.*

Essential oil	% *B. subtilis* growth inhibition ^1^ (v/v)			
	10 μL/mL	25 μL/mL	50 μL/mL	75 μL/mL
*Cinnamomum cassia*	69.7	44.4	3.9	0
*Citrus sinensis*	85.7	18.7	37.5	35.3
*Cymbopogon matinii var. motia*	79.8	50.5	50.3	54.2
*Elettaria cardamomum*	23.8	44.9	51.6	52.6
*Eugenia caryophyllus*	91.6	92.4	90.1	84.4
*Mentha arvensis*	31.7	45.2	56.3	62.3
*Mentha x piperita*	71.6	61.9	61.1	63.6
*Origanum compactum*	76.7	64.9	64.8	68.5
*Thymus vulgaris CT Linanol*	95.3	78.4	89.3	94.6
*Thymus satureioides*	78.4	78.3	80.2	84.4

^1^ Values are results of two experiments with two replicates.

### Combined antibacterial effect

After comparing the antibacterial activity of these ten EOs against *B. subtilis* using disk diffusion and broth dilution methods, and considering their antimicrobial components, we focused on *Cymbopogon matinii var. motia,* the main component of which is geraniol. Palmarosa oil, with specific rose fragrance, ^42^ appears to be a good candidate to be used as an antibacterial agent against *Bacillus subtilis* in the bakery industry. Table 5 shows the results of the synergestic antibacterial activity of *Cymbopogon matinii var. motia* combined with each of four EOs: *Eugenia caryophyllus*, *Mentha arvensis*, *Mentha x piperita* and *Thymus vulgaris CT Linalol.* For this study, we carried out disk diffusions using a 1:1 (v/v) combination for *C. martini var. mortia* and each EO selected. The results showed that three combinations of EOs with different bioactive components displayed an insignificant antibacterial activity against these Gram-positive bacteria: geraniol/menthol, geraniol/menthol-menthone, geraniol/linalool. Only the geraniol/eugenol combination showed an antagonistic effect.

**Table 5.** Interaction among essential oils against *B. subtilis* growth. Fractional inhibitory concentration and interaction among essential oil major components

Binary composition of essential oils (50% v/v)	Major chemical components	Combined antibacterial effect	Interaction ^1^
*Cymbopogon matinii var. motia/Eugenia caryophyllus*	Geraniol/Eugenol	0	A
*Cymbopogon matinii var. motia/Mentha arvensis*	Geraniol/Menthol	2.06	I
*Cymbopogon matinii var. motia/Mentha x piperita*	Geraniol/Menthol	2.15	I
*Cymbopogon matinii var. motia/Thymus vulgaris CT Linalol*	Geraniol/Linalool	1.76	I

^1^ I. indifference; A. antagonist.

## Discussion

EOs are composed of a mixture of complex, low-molecular-weight organic compounds such as terpenoids, phenolic acids, flavonoids, and phenylpropanoids. ^39^ They represent a natural source of bioactive compounds. Their constituents play a key role in antimicrobial activity, through properties which are toxic for bacteria and other microorganisms. For example, the phenolic content causes disruption of plasma membrane structure and alters the membrane permeability ^43^; terpenes and terpenoids alter the permeability of the plasma membrane when interacting with their fatty acids, allowing for the release of cytoplasmic constituents. ^7^^,^^44^ All the EOs tested showed antibacterial properties against the Gram-positive bacterium *B. subtilis* ( Table 2), some of them having a weaker antibacterial effect than others. The bioactive components 1,8-cineole (present in: ravintsara, saro or mandravasarota, *Eucalyptus officinalis*, laurel noble and niaouli oils), α-pinene (present in: cineole blueberry and verbenone rosemary oils), α-zingiberene (present in ginger oil), limonene (present in: yuzu junos, grapefruit, mandarin and sweet orange oil), sabinene (present in: ravintsara and laurel noble oil) are known for their weak antibacterial activity, compared to alcoholic and phenolic monoterpenes such as carvacrol and thymol (present in Origan compact oil). ^34^^,^^45^ The monocyclic monoterpenoid compound terpinen-4-ol has been shown to inhibit *B. cereus* biofilm formation ^46^ and *Staphylococcus aureus* ^36^ but showed a weak effect against *B. subtilis.* The antibacterial activity of the *Cymbopogon martinii var. motia* EO (palmarosa oil) could be related to its high levels of geraniol, an acyclic monoterpene alcohol. The antimicrobial activities of EOs appear to be related to their chemical composition, and our results corroborate previous studies showing their antimicrobial activity against the *Bacillus* genus from the work by Syed *et al.* ^47^

*Cinnamomum cassia* and *Origanum compactum* oils showed more activity at higher concentration (no dilution, 100%) in agar-disk diffusion assays; according to the results ( Table 2) the former EO’s antimicrobial action may be associated with the presence of high contents of E-cinnamaldehyde that can be compared with the antibacterial activity of the carvacrol and thymol present in *O. compactum* oil. This was previously described by Helander *et al.* (1998) in relation with the inhibitory activity against *Escherichia coli*. ^48^ Regarding the antimicrobial activity shown by sesquiterpenes, which are the main compounds of *Curcuma longa*, their aromatic group leads to hydrophobicity. Their poor water solubility may explain the lower antibacterial activity against *B. subtilis* as described by Tønnesen *et al.* (2002). ^49^ The antimicrobial activity of oil constituents from *C. nobile* ( Table 2) was previously described by Piccaglia *et al.* (1993) ^50^ and is known for its therapeutic uses, especially through its binding to different cell receptors involved in several biochemical pathways, related to inflammation and several metabolic disorder. ^51^ Our results showed around 26% inhibition of the growth of *B. subtilis*, when compared with the antibacterial activity of antibiotic ciprofloxacin, which acts at the level of the bacterium DNA. ^52^ *Zingiber officinale* essential oil has been used as a natural food additive and preservative, and previous studies have shown that the strongest antibacterial effect of this EO was observed against *B. subtilis* ^53^; this corroborates our results. The twenty-four EOs tested with different major chemical components ( Table 1) had different degrees of growth inhibition against *B. subtilis,* and their antibacterial activities were reduced at lower oil concentrations; according to the results ( Table 2) the inhibition halo was dependent on each essential oil.

After analysing EO antimicrobial effect using agar-disk diffusion assays, we focused on 10 EOs ( Figure 2) to evaluate their antibacterial activity in aqueous solution, measured as MIC ( Table 4). These EOs showed antimicrobial activity against *B. subtilis*, with a wide range of inhibition values; the lowest MIC values were found for cardamon, sweet orange, field mint and palmarosa oil. Low antimicrobial activity was observed for cimbru, thyme with savoury leaves, peppermint and clove bud oils. The hydrophobic characteristic of some compounds from the EOs (E-cinnamaldehyde, limonene, carvacrol, linalool, thymol, borneol) can explain the difference in antibacterial activity between broth medium culture and disk-diffusion method, for some EOs such as Chinese cinnamon and oregano compact oils.

This study has conducted an analysis about the antibacterial activity of the EOs against *B. subtilis* ( Tables 2 and 3). Taking into consideration the previous results from other authors in this matter, ^54^ we set a goal to perform a specific study for *C. martinii var. motia*, EO with a high geraniol content ( Table 1) to which different biological activities have been attributed (antimicrobiological, antioxidant and anti-inflammatory) by Mączka *et al*., ^55^ highlighting the fact that EOs contains major and minor chemical components, and their combination can contribute to their antimicrobial properties. However, due to the impact of the taste and scent of some EOs, their application as food preservatives is not fully extended; therefore, the combination of different EOs is an alternative to improve these effects as well as to reduce their organoleptic impact in food. It was suggested that some mixtures of these EOs could be determining synergistic, antagonistic, or absence of interactions between them against bacterial growth. ^56^^,^^57^ Studies on the antimicrobial activity of EO associations were developed using binary combination at the same proportion (v/v), using the disk diffusion method, which consisted of four binary combinations of *C. martinii var. motia* with *E. caryophyllus*, *T. vulgaris CT Linalol*, *M. arvensis* and *Mentha x piperita* EOs. The results of the combined effect of blending of monoterpene alcohols (geraniol, linalool), cyclic terpene alcohol (menthol), phenylpropanoids (eugenol), is showed in Table 5. The geraniol/eugenol combination showed antagonistic effects on the growth of *B. subtilis*, while geraniol/menthol and geraniol/linalool showed no effect. *Eugenia caryophyllus* did not show an antimicrobial effect when it was tested independently (2.5 μL). However, the study carried out by Galluci *et al.* (2009), ^57^ showed that the geraniol/menthol combination exhibited a high antimicrobial activity against *B. cereus*, and the synergistic antimicrobial activity of geraniol/eugenol was partially efficient against the bacteria.

Pathogen control in the food industry is the key to ensure food safety, ^58^ including bakery products, which play important roles in human health and diet. ^59^ Previous authors have also shown the antibacterial activity of monoterpenes were present in EOs, and their potential use for the food industry. ^20^^,^^25^^,^^60^ Geraniol is non-polar, making it more able to permeate the lipid structure of microorganic cell membranes, causing K ^+^ leakage from *Saccharomyces cerevisiae*. ^61^ Considering that geraniol is the principal components of *C. martinii var. motia* (> 80%), together with geranyl acetate, linalool and b-caryophyllene, it appears to be a good alternative as an additive.

Fresh dough is a type of product readily susceptible to microbial deterioration; however, many of the chemicals licensed for use as food preservatives are being questioned regarding their effects on human health. The bakery industry tries to control microbiological spoilage by following several strategies including reformulation of the product and incorporating some preservatives; this is not an easy task, as microorganisms are found in the air or in the water. There are few research reports on EO applications in bread or other bakery products, or the impact their addition can have on dough and bread production, on physico-chemical, microbiological, and taste aspects. The spoilage of bakery products may occur through microbiological contamination.

Rope formation is a serious, but underreported food security problem in the bakery industry. Although this problem has been recognized for many years, effective means of prevention have not yet been determined. *B. subtilis* is one the bacteria responsible for rope spoilage in bread preservation processes. ^62^^,^^63^ Recent studies have revealed the antibacterial activity of different EOs applied in bakery products including thyme, cinnamon, oregano, and lemongrass, that can inhibit the growth of harmful microorganisms, resulting in a product with extended shelf-life and enhanced safety. ^63^ Palmarosa oil is a good alternative as an additive in the bakery industry since in addition to its antimicrobial activity, its volatility has been previously shown to cause the reduction of approximately 60% of the geraniol component over 24 hours. ^61^ A controlled liberation of the EO and the high evaporation rate of geraniol may avoid lethal damage of baking yeast during the bread-making process.

Nanotechnologies offer very interesting prospects for food industry, the 'novel food' includes innovative food, as well as food produced using new technologies and production processes. References to nanotechnology and nanomaterials in European regulations are scarce, especially to the use of essential oils as preservatives for bakery products. ^64^ The use of palmarosa oil could be considered to avoid the presence of “rope in bread”, considering their activity against *B. subtilis* and its pleasant fragrance, which can improve the flavor of the product. At the same time, the palmarosa essential oil plays an important role as an antioxidant in food, thus preventing potential health risks associated with microbial contamination. It is highly reactive against free radicals from reactive oxygen species generated by a wide variety of sources in biological systems. ^65^ Microencapsulation using EOs presents the advantage that it maintains the effectiveness of antimicrobial activity through the gradual release of the active components of EO, from the capsules to the bakery product. We must not ignore other possible uses of palmarosa oil, and its high proportions of geraniol, to increase the safety of bakery products, such as essential oil-loaded films, analysed by Agarwal *et al*. (2020), ^66^ which can be used in active bakery ingredients such as those used in sourdough.

## Conclusions

The antimicrobial activity of EOs depends on their composition in volatile compounds, such as terpenes, terpenoids, phenol-derived aromatic components and aliphatic components. They represent a natural source of bioactive compounds. A total of 24 EOs, with different compositions, have been analysed.

The EOs tested showed different antibacterial effects against the growth of *Bacillus subtilis.* These antibacterial activities were reduced at lower oil concentrations; thus, the result from the disk diffusion tests suggest that the antibacterial activity of each EO is dose-dependent. These results show the following antimicrobial activity in order of efficiency in agar diffusion disk assays: phenylpropene > terpenoid > alcohol terpene > terpene > sesquiterpene. It was found that the oils containing alcohol, ketone, ester groups and hydrocarbon as major constituents exhibited a greater antimicrobial activity, whereas the oils containing aldehyde or methoxy groups covalently linked to aromatic organic compounds such as phenyl and phenol groups, exhibited dominant diffusion activity.

The results obtained in this study confirm that *Cymbopogon martinii var. motia*, which contains geraniol, a compound with antioxidant effects, may be used to prevent the growth of *B. subtilis,* responsible for “rope formation” in the bakery industry. The formulation of palmarosa/clove bud EOs tested in this study have an antagonistic effect against the growth of *B. subtilis.*

The possible use of palmarosa oil, as a potential natural solution to increase the shelf life and safety of bakery products, brings new technological solutions. With the development of techniques such as nanoencapsulation for bakery doughs, active packaging of baked products or new surface disinfectants, the *Cymbopogon martinii var. motia* essential oil can be an alternative in the bakery industry due to his high evaporation rate and organoleptic effect.

## Data availability

### Underlying data

Figshare: Antibacterial activity of EO against Bacillus subtilis, https://doi.org/10.6084/m9.figshare.15129057. ^67^

Data are available under the terms of the Creative Commons Zero “No rights reserved” data waiver (CC BY 4.0 Public domain dedication).
