# Supplementary material for: Essential oil of Cymbopogon martini, source of geraniol, as a potential antibacterial agent against Bacillus subtilis, a pathogen of the bakery industry
Source: F1000Res. 2023 Mar 24;10:1027. Originally published 2021 Oct 11. [Version 2] doi: 10.12688/f1000research.54196.2 (PMC9936101; doi:10.12688/f1000research.54196.2)
Supplement: Peer review history

# Peer review: Reviewer response for version 2

**DOI:** 10.5256/f1000research.144745.r167701

**Published:** 2023-03-27

**Authors:** Daniel Rico

**Affiliations:** 1 Agrarian Technological Institute of Castilla and Leon (ITACyL), Valladolid, Spain

I have no further comments. All questions raised in my previous report have been accordingly answered by the authors.

Is the work clearly and accurately presented and does it cite the current literature?

Yes

If applicable, is the statistical analysis and its interpretation appropriate?

Yes

Are all the source data underlying the results available to ensure full reproducibility?

Yes

Is the study design appropriate and is the work technically sound?

Yes

Are the conclusions drawn adequately supported by the results?

Yes

Are sufficient details of methods and analysis provided to allow replication by others?

Yes

Reviewer Expertise:

Food processing (novel technologies). Postharvest. Bioactivity (in vitro) determination of food matrices and ingredients.

I confirm that I have read this submission and believe that I have an appropriate level of expertise to confirm that it is of an acceptable scientific standard.

# Peer review: Reviewer response for version 1

**DOI:** 10.5256/f1000research.57656.r160708

**Published:** 2023-02-16

**Authors:** Abubakar Sunusi Adam

**Affiliations:** 1 Federal University Dutsin-Ma (FUDMA),,, Katsina State, Nigeria

Details of some methods have not been fully addressed. For example, the author stated that they used nutrient broth agar plate for antibacterial activity of the essential oil which is not appropriate. As such, the author should know that the standard medium to be used for any sensitivity testing is Mueller Hinton agar. However, broth and agar are two different entity, both cannot be use concurrently.

 Furthermore, preparation of essential oil concentrations was not clearly explained.  

 Under discussion, the author should compare the current findings with related reports from other researchers. This is because, different bacteria have different response against antimicrobial agents, in which essential oils are inclusive.

 Otherwise, the authors did a good job.

Is the work clearly and accurately presented and does it cite the current literature?

Yes

If applicable, is the statistical analysis and its interpretation appropriate?

I cannot comment. A qualified statistician is required.

Are all the source data underlying the results available to ensure full reproducibility?

Yes

Is the study design appropriate and is the work technically sound?

Yes

Are the conclusions drawn adequately supported by the results?

Yes

Are sufficient details of methods and analysis provided to allow replication by others?

Yes

Reviewer Expertise:

Antimicrobial resistance

I confirm that I have read this submission and believe that I have an appropriate level of expertise to confirm that it is of an acceptable scientific standard.

# Response

**Published:** 2023-02-21

**Authors:** Maria Angeles Rojo

**Affiliations:** Miguel de Cervantes European University, Spain

Thanks for your advice and corrections. Below we provide responses to your observations.

*1. “For example, the author stated that they used nutrient broth agar plate for antibacterial activity of the essential oil which is not appropriate. As such, the author should know that the standard medium to be used for any sensitivity testing is Mueller Hinton agar”*

 The remark proposed is right. In the present study “Mueller Hinton agar” was considered to be a non-selective medium; the purpose of our study was to analyze EOs activity under the same conditions as those where the bacteria were laboratory-grown and preserved. Commercial EOs were not to be filtered prior to their use on the disk diffusion microbioassay in order to keep their small-sized volatile components. Yet, it would be of the utmost interest to conduct the same trials concerning the biological activity in the standard medium to compare the outcomes regarding antimicrobial activity.

*2. “However, broth and agar are two different entity”*

 It has been corrected in the text.

*3. “preparation of essential oil concentrations was not clearly explained”*

 Thank you for your comment, it has been modified in the text. Furthermore, it can also be stated that we used the microdilution method, where essential oils were 10 or 100 fold diluted. The % unit  was used to indicate the volume of EO added.

*4. “Under discussion, the author should compare the current findings with related reports from other researchers. This is because, different bacteria have different response against antimicrobial agents, in which essential oils are inclusive”*

 In the bibliography we located studies of the same bacterium under study where the authors searched for antagonistic microorganisms against *B. subtillis *[Pepe O, Blaiotta G, Moschetti G, Greco T, Villani F. Rope-producing strains of Bacillus spp. from wheat bread and strategy for their control by lactic acid bacteria. Appl Environ Microbiol. 2003 Apr;69(4):2321-9].

 In view to the study of ropiness in bread, *B. subtillis* is the bacteria that, according to our preliminary bibliographic search, was the least used in the analysis against the action of EOs.

 Concerning the characteristics *of C. martinii *var. *motia,* no similar studies were found. Just to mention an example, a bacteria analyzed by different authors is *S. aureus* due to its production of different toxins and its implication in human health.

# Peer review: Reviewer response for version 1

**DOI:** 10.5256/f1000research.57656.r156436

**Published:** 2023-01-10

**Authors:** Daniel Rico

**Affiliations:** 1 Agrarian Technological Institute of Castilla and Leon (ITACyL), Valladolid, Spain

The work titled “Essential oil of *Cymbopogon martini*, source of geraniol, as a potential antibacterial agent against *Bacillus subtilis*, a pathogen of the bakery industry”, and submitted for publication to the F1000Research journal, is focused on screening the antibacterial effect against *B. subtilis* of a number of chemotyped essential oils.

 The experimental design is appropriate to the objective intended, and the experimental methodology sufficiently described in detail. Antimicrobial activity of a significant number of essential oils against *B. subtilis* is presented, and discussed based on the main components present in these EOs. Interesting potential of the *Cymbopogon martinii* var. *motia* essential oil is concluded by the authors.

 I find the work suitable for indexing after minor revision. Please check comments below.

 Section Results/Chemical composition of EOs (Pranarôm [S.A]). Since the results showed in this subsection are a compilation of the information provided by the essential oil manufacturer, I would recommend the authors to reduce the extension of this part, and keep the minimal extension to fulfil the main purpose of classification of the different EOs used based on their main compound. I would also recommend to avoid the use of terms such as “analysed” or “identified”, which could create some confusion on the origin of the results of this subsection.

 Subsection “Determination of minimum inhibitory concentration of essential oils against *B. subtilis*” (in Methods, Page 4). Regarding EOs selection of 10 for the second part of the experimental work, the authors have stated that “This selection also considered the principal chemical component in their composition”. Could the authors please be more specific and clarify the criteria for this selection of 10 EOs? Many thanks.

 In table 2, the description given of the second calculated parameter, “% *B. subtilis* growth inhibition against Ciprofloxacin”, seems a bit confusing. Please provide a clearer definition, as it appears that a 0% should correspond to no inhibition at all, as it says growth inhibition, when from the equation and diameters of inhibition areas, it appears 0% corresponds to 100% inhibition (similar to ciprofloxacin).

 Please specify in material and methods, section “Combined antibacterial effect of EOs using disk diffusion test”, how values over 1 are classified, as some over 1 are shown in Table 4, and classified as “indifference”

 Section “Conclusion”. It would be interesting to add some comments in regard the concentration of the EO that should be necessary for the applications (bakery) suggested.

 Also, a comment on one of the sentences in this same section. Please revise the sentence finishing “…groups, exhibited dominant diffusion activity”, and check if it conveys the intended meaning by the authors. Maybe the word “dominant” should be checked. Thank you.

Is the work clearly and accurately presented and does it cite the current literature?

Yes

If applicable, is the statistical analysis and its interpretation appropriate?

Yes

Are all the source data underlying the results available to ensure full reproducibility?

Yes

Is the study design appropriate and is the work technically sound?

Yes

Are the conclusions drawn adequately supported by the results?

Yes

Are sufficient details of methods and analysis provided to allow replication by others?

Yes

Reviewer Expertise:

Food processing (novel technologies). Postharvest. Bioactivity (in vitro) determination of food matrices and ingredients.

I confirm that I have read this submission and believe that I have an appropriate level of expertise to confirm that it is of an acceptable scientific standard.

# Response

**Published:** 2023-02-21

**Authors:** Maria Angeles Rojo

**Affiliations:** Miguel de Cervantes European University, Spain

Thanks for your advice and corrections. Below we provide responses to your observations.

*1. “Section Results/Chemical composition of EOs (Pranarôm [S.A]). Since the results showed in this subsection are a compilation of the information provided by the essential oil manufacturer, I would recommend the authors to reduce the extension of this part, and keep the minimal extension to fulfil the main purpose of classification of the different EOs used based on their main compound. I would also recommend to avoid the use of terms such as “analysed” or “identified”, which could create some confusion on the origin of the results of this subsection”.*

 The information in the section of results has been reduced. The terms "analysed" or "identified" have been replaced by "assayed" and "evaluated" respectively.

*2. Subsection “Determination of minimum inhibitory concentration of essential oils against B. subtilis” (in Methods, Page 4). Regarding EOs selection of 10 for the second part of the experimental work, the authors have stated that “*

 This selection also considered the principal chemical component in their composition”. Could the authors please be more specific and clarify the criteria for this selection of 10 EOs? “In the section we have expanded the information which, in addition to taking into account our results, considers previously published results

*3. “In table 2, the description given of the second calculated parameter, “% B. subtilis growth inhibition against Ciprofloxacin”, seems a bit confusing. Please provide a clearer definition, as it appears that a 0% should correspond to no inhibition at all, as it says growth inhibition, when from the equation and diameters of inhibition areas, it appears 0% corresponds to 100% inhibition (similar to ciprofloxacin).”*

 The appreciation has been considered and we have changed "% B. subtilis growth inhibition against Ciprofloxacin" to " Inhib itory activity of EOs against Ciprofloxacin (% ) ". In addition, a footnote has been added referring to equation 1 in the methods section.

*4. “Please specify in material and methods, section “Combined antibacterial effect of EOs using disk diffusion test”, how values over 1 are classified, as some over 1 are shown in Table 4, and classified as “indifference””*

*In the methodology section, it has been described in the wrong way. And therefore, the calculations reflected in Table 4 are erroneous.*

 They have been corrected

*Actually, the combination effect is calculated: I=D/(DA+DB)*

 Thank you, It has been corrected

*5. “Section “Conclusion”. It would be interesting to add some comments in regard the concentration of the EO that should be necessary for the applications (bakery) suggested.”*

 Thank you for your suggestion. In our study we only focused on the possibility of using EO for bakery, analysing its effectiveness on the growth of B. subtilis. But the proposal is interesting to extend the study, our group is in fact extending the analysis to bakery products.

*6. “Also, a comment on one of the sentences in this same section. Please revise the sentence finishing “…groups, exhibited dominant diffusion activity”, and check if it conveys the intended meaning by the authors. Maybe the word “dominant” should be checked. Thank you.”*

 Thank you for your correction, it has been added “activity in disk diffusion method”
